# Protective Role of Taurine on Rat Offspring Hypertension in the Setting of Maternal Chronic Kidney Disease

**DOI:** 10.3390/antiox12122059

**Published:** 2023-11-29

**Authors:** You-Lin Tain, Chih-Yao Hou, Guo-Ping Chang-Chien, Sufan Lin, Chien-Ning Hsu

**Affiliations:** 1 Division of Pediatric Nephrology, Kaohsiung Chang Gung Memorial Hospital, Kaohsiung 833, Taiwan; tainyl@cgmh.org.tw; 2 Institute for Translational Research in Biomedicine, Kaohsiung Chang Gung Memorial Hospital, Kaohsiung 833, Taiwan; 3 College of Medicine, Chang Gung University, Taoyuan 330, Taiwan; 4 Department of Seafood Science, National Kaohsiung University of Science and Technology, Kaohsiung 811, Taiwan; chihyaohou@webmail.nkmu.edu.tw; 5 Institute of Environmental Toxin and Emerging-Contaminant, Cheng Shiu University, Kaohsiung 833, Taiwan; guoping@csu.edu.tw (G.-P.C.-C.); linsufan2003@csu.edu.tw (S.L.); 6 Center for Environmental Toxin and Emerging-Contaminant Research, Cheng Shiu University, Kaohsiung 833, Taiwan; 7 Super Micro Mass Research and Technology Center, Cheng Shiu University, Kaohsiung 833, Taiwan; 8 Department of Pharmacy, Kaohsiung Chang Gung Memorial Hospital, Kaohsiung 833, Taiwan; 9 School of Pharmacy, Kaohsiung Medical University, Kaohsiung 807, Taiwan

**Keywords:** taurine, developmental origins of health and disease (DOHaDs), nitric oxide, renin–angiotensin–aldosterone system (RAAS), chronic kidney disease, gut microbiota, hypertension

## Abstract

Taurine is a natural antioxidant with antihypertensive properties. Maternal chronic kidney disease (CKD) has an impact on renal programming and increases the risk of offspring hypertension in later life. The underlying mechanisms cover oxidative stress, a dysregulated hydrogen sulfide (H_2_S) system, dysbiotic gut microbiota, and inappropriate activation of the renin–angiotensin–aldosterone system (RAAS). We investigated whether perinatal taurine administration enables us to prevent high blood pressure (BP) in offspring complicated by maternal CKD. Before mating, CKD was induced through feeding chow containing 0.5% adenine for 3 weeks. Taurine was administered (3% in drinking water) during gestation and lactation. Four groups of male offspring were used (*n* = 8/group): controls, CKD, taurine-treated control rats, and taurine-treated rats with CKD. Taurine treatment significantly reduced BP in male offspring born to mothers with CKD. The beneficial effects of perinatal taurine treatment were attributed to an augmented H_2_S pathway, rebalance of aberrant RAAS activation, and gut microbiota alterations. In summary, our results not only deepen our knowledge of the mechanisms underlying maternal CKD-induced offspring hypertension but also afford us the impetus to consider taurine-based intervention as a promising preventive approach for future clinical translation.

## 1. Introduction

An estimated one in three adults across the earth live with hypertension [1]. Considering reducing hypertension remains a global public health problem, the WHO released guidelines on the treatment of hypertension in 2021 [2]. Most efforts, however, have gone into treating hypertension rather than keeping it from happening [3]. Noteworthy is that hypertension can originate in early life [4,5]. Hypertension is being included in the research of developmental origins of health and disease (DOHaDs) [6]. Various environmental factors occurring during the early stages of life can permanently alter organ structure and function, which leads to the developmental programming of hypertension when individuals reach adulthood [4,5,6,7]. Since early intervention may reverse adverse programming processes via reprogramming [4], the underlying mechanisms of developmental programming could be a therapeutic target for the prevention of hypertension.

The developing kidney is intrinsically susceptible to adverse early life environments, leading to long-lasting changes in morphology and function, known as so-called renal programming [7]. Renal programming has a decisive role in hypertension of developmental origins [6,7]. Oxidative stress is implicated in the pathogenesis of renal programming, which has a key role in the development of hypertension [8]. Increasing evidence suggests that effectively using perinatal natural antioxidants enables one to avert hypertension of developmental origins in several animal models [9]. Among various nutrients for the human body, natural antioxidants have received great attention over the past decades.

Taurine is a sulfur-containing amino acid with antioxidant properties [10]. The antioxidant actions of taurine cover reducing superoxide generation, enhancing antioxidant enzyme activity, stabilizing respiratory chain complexes, and inhibiting mitochondria-mediated apoptosis [11]. Additionally, taurine has some possibly advantageous effects that implicate regulation of the hydrogen sulfide (H_2_S) signaling pathway, the nitric oxide (NO) system, the renin–angiotensin–aldosterone system (RAAS), and gut microbiome homeostasis [11,12,13,14]. Although the antihypertensive effects of taurine have been studied in several human and animal studies [12], relatively little is known about its reprogramming effect against offspring hypertension [15,16].

In chronic kidney disease (CKD), taurine concentrations are decreased [17]. Previously, our study revealed that maternal CKD caused offspring hypertension accompanied by oxidative stress and gut microbiota dysbiosis [18], but it is unclear whether perinatal taurine administration can avert hypertension in adult offspring complicated by maternal CKD. Considering the link between H_2_S, NO, RAS, and gut microbiota behind hypertension of developmental origins [6,19,20], it will be of interest to determine whether they play roles in the protective mechanisms underlying taurine. In this study, we examined the effects of perinatal taurine administration on offspring’s BP and elucidated the underlying protective mechanisms using a rat model of adenine-induced maternal CKD.

## 2. Materials and Methods

### 2.1. Animal Model

Sprague–Dawley (SD) rats were purchased from BioLASCO Taiwan Co., Ltd. (Taipei, Taiwan). The animal housing facility was maintained on a 12:12 h light–dark cycle with free access to food and water. All procedures followed Animal Research: Reporting of In Vivo Experiments (ARRIVE) guidelines and were reviewed by the IACUC of Chang Gung Memorial Hospital (protocol no. 2022061301).

**Figure 1:**
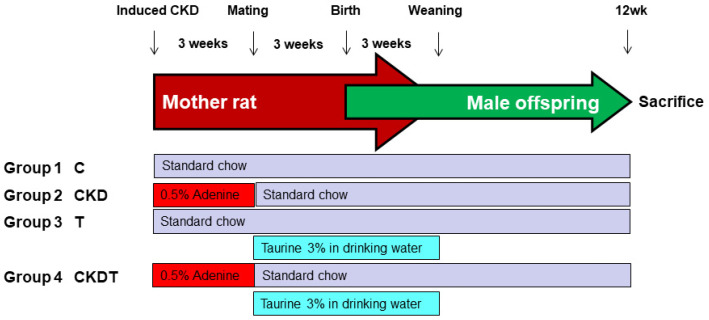
Experimental design and animal grouping.

As outlined in Figure 1, to conduct a CKD model, female rats were provided a standard chow (control group) or a chow containing 0.5% adenine (Merck KGaA, Darmstadt, Germany) during 8–11 weeks of age [18]. At 11 weeks of age, female rats were bred with the males. The presence of the plug confirmed mating. A total of 12 dams were randomly allocated to one of four groups (*n* = 3 per group): the C group (sham control), the CKD group (adenine-fed rats), the T group (control rats which received 3% taurine in drinking water during pregnancy and lactation), and the CKDT group (adenine-fed rats which received taurine treatment). The dose of taurine utilized here was based on previous rat research [15]. The litter size was restricted to eight pups. Because females were protected from developing hypertension [21], we only enrolled 8 male offspring from each group for use in subsequent experiments.

BPs were measured using a CODA non-invasive BP system (a tail-cuff method, Kent Scientific Corporation, Torrington, CT, USA). At 12 weeks of age, offspring rats were killed. Stool samples were collected and stored at −80 °C until analysis. Plasma samples were collected in tubes containing heparin. We collected kidney samples via dividing the cortex and medulla, snap-freezing, and storing at −80 °C in a freezer.

### 2.2. Analysis of H_2_S-Generating Enzymes and RAAS Components using qPCR

RNAs isolated from renal cortical tissues were processed for quantitative real-time polymerase chain reaction (qPCR) using a Bio-Rad iCycler iQ real-time PCR detection system (Bio-Rad, Hercules, CA, USA) in duplicate. Four H_2_S-generating enzymes were analyzed, including cystathionine γ-lyase (CSE), cystathionine β-synthase (CBS), d-amino acid oxidase (DAO), and 3-mercaptopyruvate sulphurtransferase (3MST).

We measured numerous RAAS components, including renin, (pro)renin receptor (PRR), angiotensin converting enzyme (ACE), angiotensinogen (AGT), and angiotensin II type 1 receptor (AT1R). We used the 18S ribosomal RNA (R18S) as the reference gene. The primers are provided in Table 1. The relative gene expression was calculated based on the comparative CT method. Fold-differences were calculated using the formula 2^−ΔΔCT^.

### 2.3. Tissue H2S-Producing Capacity

Renal H_2_S-producing capacity was analyzed as described previously [22]. Briefly, kidney cortex tissues (*w*/*v*, 1:10) were homogenized in 100 mM ice-cold potassium phosphate buffer (pH 7.4). Tissue homogenates were then incubated with 10 mM L-cysteine, 2 mM pyridoxal 5′-phosphate, and saline in sealed Eppendorf vials for half an hour. Later, zinc acetate (1% *w*/*v*, 250 μL) was injected, followed by trichloroacetic acid (10% *w*/*v*, 250 μL) to precipitate proteins and stop the reaction. Then, *N*,*N*-dimethyl-p-phenylenediamine sulfate in 7.2 M HCl and FeCl_3_ in 1.2 M HCl were added. After 15 min, the absorbance (670 nm) of the resulting solution was measured. The calibration curve of sodium hydrosulfide (3.125–250 μM) was utilized to determine the H_2_S concentration in each sample. All of the buffers and chemicals were purchased from Sigma-Aldrich (St. Louis, MO, USA) and Thermo Scientific (Waltham, MA, USA).

### 2.4. Analysis of NO Parameters Using HPLC

NO-related parameters include L-citrulline (the precursor of L-arginine), L-arginine (the substrate for NO synthase), and asymmetric and symmetric dimethylarginine (ADMA and SDMA, inhibitors of NO synthase). Their concentrations were analyzed with an Agilent Technologies Series 1100 HPLC (Santa Clara, CA, USA) with an o-phthaldialdehyde/3-mercaptopropionic acid (OPA-3MPA; Sigma-Aldrich, St. Louis, MO, USA) derivatization reagent according to our formerly validated protocol [22].

### 2.5. 16S rRNA Sequencing

Genomic DNA of the stool samples from rat offspring was subjected to 16S rRNA sequencing at Biotools Co., Ltd. (New Taipei City, Taiwan) [23]. The full-length 16S genes covering the V1–V9 region were amplified with barcoded primers for multiplexed SMRTbell library (PacBio, Menlo Park, CA, USA) preparation and sequencing procedure. A QIIME2 phylogeny fast tree was applied to create a phylogenetic tree with a set of sequences representative of the amplicon sequence variants (ASVs) [24]. We analyzed the alpha and beta diversity for bacterial communities. The alpha diversity was determined using the Pielou’s evenness and Shannon index. We assessed the β-diversity using the partial least squares discriminant analysis (PLS-DA) and the Analysis of similarities (ANOSIM). Furthermore, linear discriminant analysis effect size (LEfSe) was utilized to identify the differentially abundant taxa with LDA > 4.

### 2.6. Statistics

All data are presented as means ± the standard error of the mean (SEM). Statistical analyses were carried out via one-way ANOVA or two-way ANOVA where appropriate. If the ANOVA indicated a significant interaction between factors, statistical differences between groups were explored using the Tukey post hoc test. For all statistical comparisons, *p* < 0.05 was considered statistically significant. Statistical analysis was performed by (SPSS Inc., Chicago, IL, USA).

## 3. Results

### 3.1. Body Weight and BP

As shown in Table 2, at 12 weeks of age, body weight was lower in the CKDT group than in the other groups. The kidney weight and the ratio of kidney-weight-to-body-weight was highest in CKD offspring among the four groups. However, plasma concentrations of creatinine were not different between the four groups.

**Figure 2:**
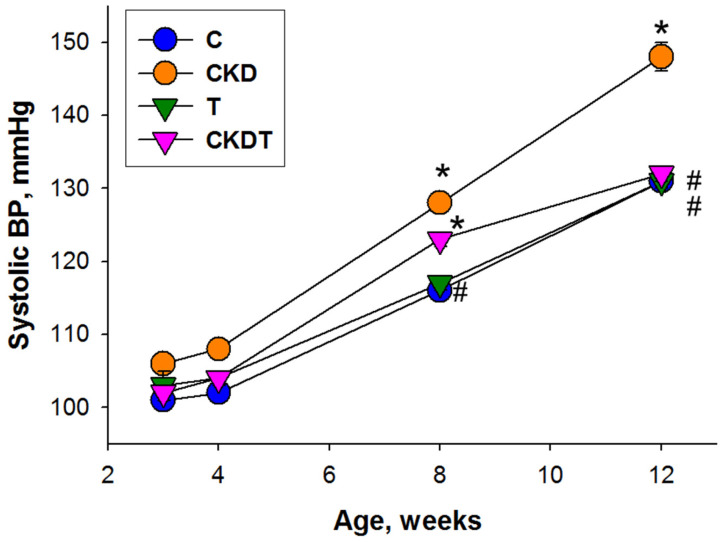
Systolic blood pressure in offspring from 3 to 12 weeks of age (*n* = 8/group). * *p* < 0.05 vs. C; # *p* < 0.05 vs. CKD. C = control offspring rats; CKD = adenine-exposed offspring rats; T = offspring rats born to dams which received taurine; CKDT = offspring rats born to adenine-treated dams which received taurine.

Longitudinal measurement of BP from weeks 3 to 12 demonstrated that maternal CKD raised offspring’s systolic BP during 8–12 weeks of age, which was restored with maternal taurine treatment (Figure 2). As also shown in Table 2, at 12 weeks of age, diastolic BP and mean arterial pressure were greater in the CKD group than in the other groups. Taken together, these observations revealed that maternal CKD resulted in hypertension and increased kidney weight in adult offspring, which maternal taurine treatment prevented.

### 3.2. H_2_S System in the Kidneys

**Figure 3:**
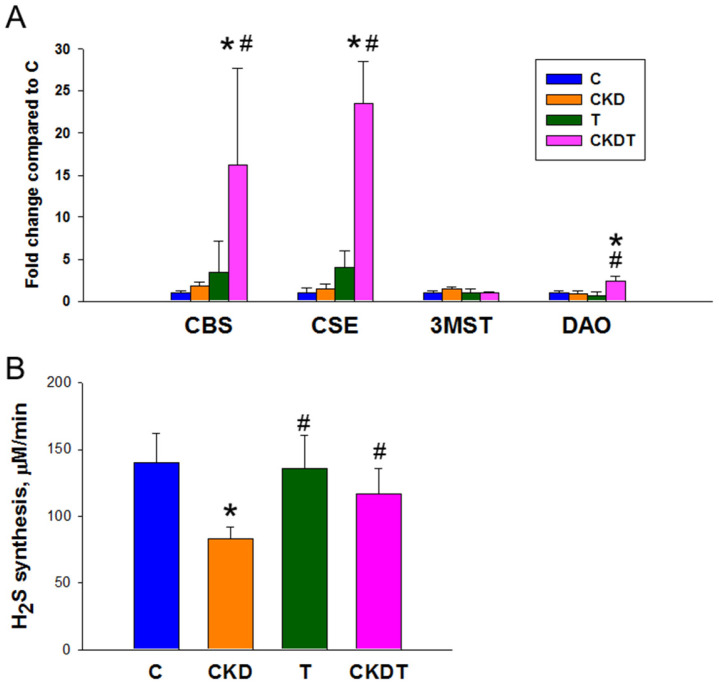
(**A**) Renal gene expression of H_2_S-generating enzymes cystathionine β-synthase (CBS), cystathionine γ-lyase (CSE), 3-mercaptopyruvate sulphurtransferase (3MST), and d-amino acid oxidase (DAO). (**B**) Renal H_2_S synthesis. * *p* < 0.05 vs. C; # *p* < 0.05 vs. CKD. C = control offspring rats; CKD = adenine-exposed offspring rats; T = offspring rats born to dams which received taurine; CKDT = offspring rats born to adenine-treated dams which received taurine.

Since four H_2_S-producing enzymes were expressed in the kidney, we next studied the gene expression of CBS, CSE, 3MST, and DAO in offspring kidneys (Figure 3A). As shown in Figure 3A, renal expression of four H_2_S-producing enzymes was comparable between the C and CKD groups. Nevertheless, taurine treatment significantly enhanced renal expression of CBS, CSE, and DAO in the CKDT group. We further analyzed H_2_S synthesis in the offsprings’ kidneys. As illustrated in Figure 3B, maternal CKD caused a reduction in renal H_2_S production, which was prevented by perinatal taurine supplementation.

### 3.3. NO Parameters

We next investigated the plasma concentrations of NO-related parameters. Table 3 illustrates that both maternal CKD and taurine treatment had a negligible effect on plasma concentrations of L-citrulline, L-arginine, ADMA, and SDMA, and the ratio of L-arginine to ADMA as well.

### 3.4. RAAS

**Figure 4:**
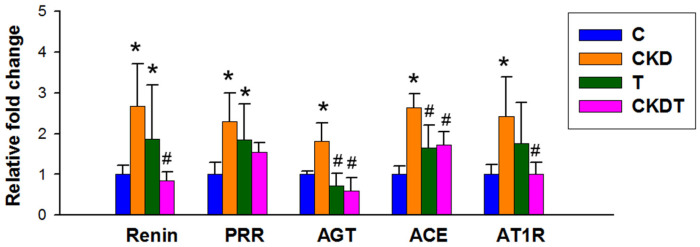
Renal gene expression of renin-angiotensin-aldosterone system components, including renin, (pro)renin receptor (PRR), angiotensinogen (AGT), angiotensin converting enzyme (ACE), and angiotensin II type 1 receptor (AT1R). * *p* < 0.05 vs. C; # *p* < 0.05 vs. CKD. C = control offspring rats; CKD = adenine-exposed offspring rats; T = offspring rats born to dams which received taurine; CKDT = offspring rats born to adenine-treated dams which received taurine.

We further assessed the RAAS using qPCR (Figure 4). Maternal CKD augmented the renal gene expression of AGT, renin, PRR, ACE, and AT1R. Renal expression of renin and PRR were higher in the T group compared with that in the C group. Maternal taurine treatment significantly reduced CKD-induced increases in renin, AGT, ACE, and AT1R expression.

### 3.5. Gut Microbiota Composition

**Figure 5:**
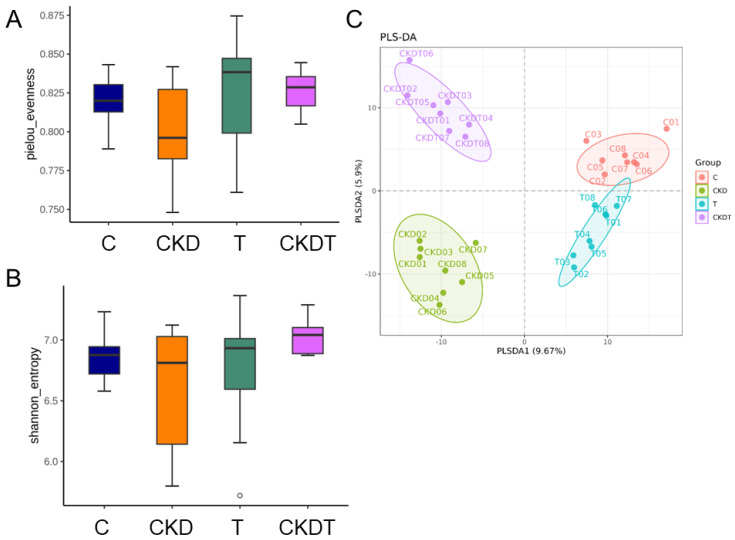
Alpha and beta diversities among groups. Alpha diversity was significant for (**A**) Pielou’s evenness and (**B**) Shannon index. *p* < 0.05 vs. C. (**C**) Partial least squares discriminant analysis (PLS-DA) plots of beta diversity. Each dot represents the microbiota of a single sample, and dots of the same color belong to the same group. C = control offspring rats; CKD = adenine-exposed offspring rats; T = offspring rats born to dams which received taurine; CKDT = offspring rats born to adenine-treated dams which received taurine.

The results on the alpha diversity index of the gut microbiota in rats showed no difference between the four groups (Figure 5A,B). However, changes were observed for beta diversity indices in the microbiota of rats. As shown in Figure 5C, fecal microbiota for the four groups can be clearly separated by PLS-DA. Additionally, there were significant differences between each group for ANOSIM (All *p* < 0.05). Our data indicated that maternal CKD and taurine treatment both led to distinct offspring enterotypes. Consistent with previous research [18,22,23], we observed that *Firmicutes* and *Bacteroidetes* were the predominant phyla.

**Figure 6:**
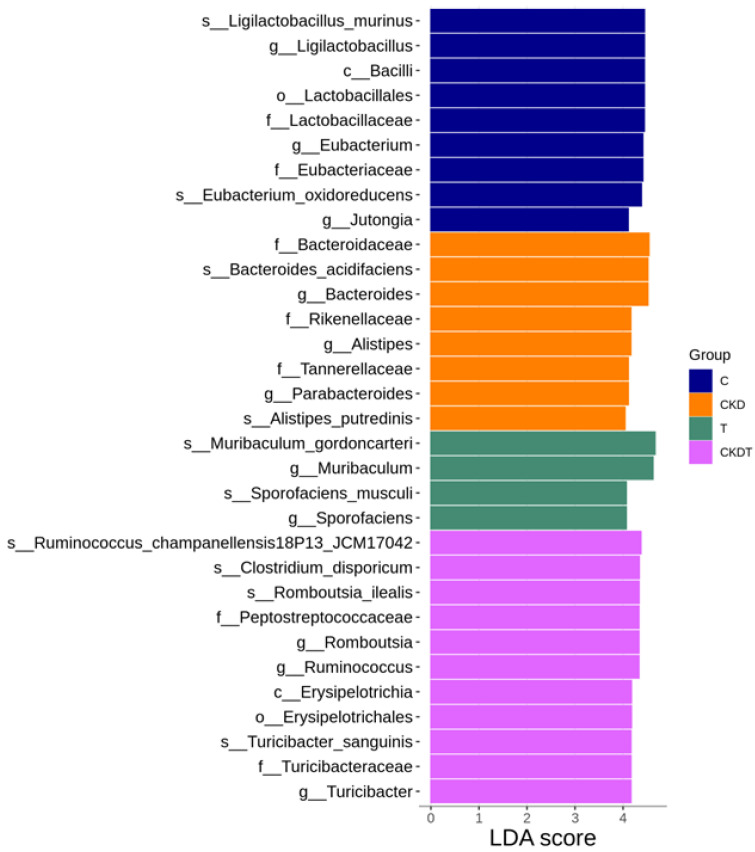
Linear discriminant analysis effect size (LEfSe) identified most differential taxa where LDA score thresholds > 4 were listed. C = control offspring rats; CKD = adenine-exposed offspring rats; T = offspring rats born to dams which received taurine; CKDT = offspring rats born to adenine-treated dams which received taurine.

We used the LEfSe algorithm to determine whether any taxa at different taxonomic levels were enriched associated with CKD and taurine treatment (Figure 6). The genera *Bacteroides*, *Alistipes*, and *Parabacteroides* were over-represented in the CKD group, while the species *Ligilactobacillus murinus* and the genus and class to which it belongs were enriched in the control group. In addition, taurine treatment resulted in a higher number of genera *Muribaculum* and *Sporofaciens* in the T group. Figure 6 also reveals that taurine supplementation caused higher levels of the genera *Romboutsia*, *Ruminococcus*, and *Turicibacter* in the CKDT group.

**Figure 7:**
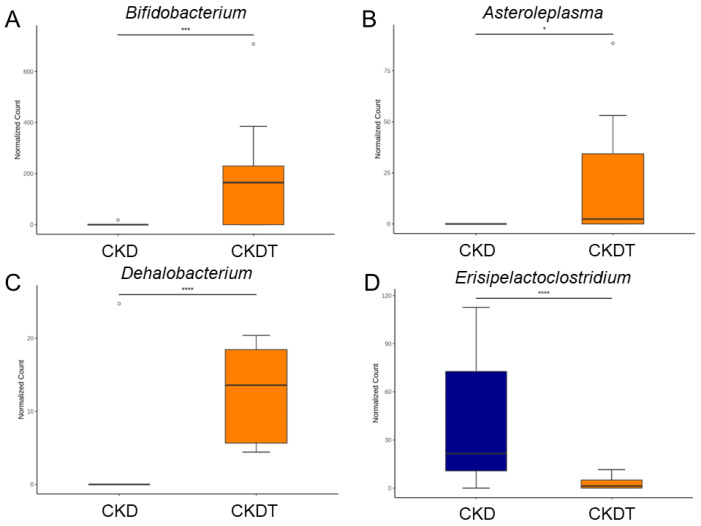
Genus-based comparison between the CKD and CKDT groups. Relative abundance of (**A**) *Bifidobacterium*, (**B**) *Asteroleplasma*, (**C**) *Dehalobacterium*, and (**D**) *Erisipelactoclostridium*. The outliers are shown as dots. * *p* < 0.05; *** *p* < 0.005; **** *p* < 0.001. CKD = adenine-exposed offspring rats; CKDT = offspring rats born to adenine-treated dams which received taurine.

A genus-based comparison showed that the proportion of *Bifidobacterium*, *Asteroleplasma*, and *Dehalobacterium* were augmented by perinatal taurine treatment in the CKDT group compared with that in the CKD group (Figure 7A–C). On the contrary, the CKDT group had a lower number of genus *Erisipelactoclostridium* vs. the CKD group (Figure 7D).

## 4. Discussion

This study shows the protective role of taurine in the developmental origins of hypertension as induced by maternal CKD during early life. Previously, we showed that at 12 weeks of age, adult offspring born to mothers with CKD had elevated BP along with increased kidney weight [18]. Although taurine has shown BP-lowering effects in prior research [12], our study is the first to demonstrate that maternal taurine administration protects adult offspring against the elevation of BP programmed by maternal CKD.

**Figure 8:**
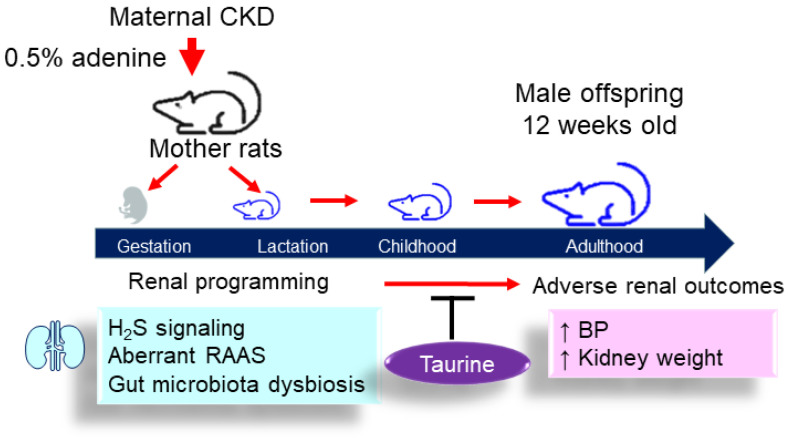
Schematic diagram summarizing the protective effects of perinatal taurine supplementation in male offspring born to dams with CKD and putative mechanisms. CKD = chronic kidney disease; H_2_S = hydrogen sulfide; RAAS = renin–angiotensin–aldosterone system; BP = blood pressure.

The key findings of the current study are as follows: (1) perinatal taurine treatment targets renal programming to halt its adverse programming processes, helping to protect adult offspring against hypertension; (2) perinatal taurine supplementation increases the gene expression of H_2_S-producing enzymes and H_2_S production in offsprings’ kidneys; (3) taurine protection against offspring hypertension coincides with the restoration of CKD-induced aberrant RAAS activation, characterized by decreases in renin, AGT, ACE, and AT1R expression; (4) maternal CKD and taurine treatment, either individually or in combination, differentially alter offsprings’ gut microbiota profile, resulting in distinct enterotypes; (5) the beneficial effect of taurine is connected with an enhanced amount of the genera *Bifidobacterium*, *Asteroleplasma*, and *Dehalobacterium* and a decrease in *Erisipelactoclostridium*. A schematic summarizing the main findings is presented in Figure 8.

In support of pregnant women with CKD who are at risk of adverse maternal and offspring outcomes [25,26], our results indicate that adult male offspring born to adenine-fed dams display hypertension and increased kidney weight, an early feature of CKD. Although plasma creatinine concentrations remain not yet elevated, the kidney weight and the kidney-weight-to-body-weight ratio are increased in the CKD offspring. Increased kidney weight could be due to renal hyperplasia or hypertrophy. The increased kidney-weight-to-body-weight ratio was used as an indicator of renal hypertrophy [27]. As renal hypertrophy can cause glomerular sclerosis, the long-term kidney outcomes of CKD offspring are worthy of additional investigation.

While taurine can have direct effects on BP [12], results from this study indicate that it also displays reprogramming effects against offsprings’ BP. One protective mechanism by which perinatal taurine protects adult offspring against hypertension is attributed to augmentation of the H_2_S pathway. Previously, our work revealed that the use of precursors of H_2_S, such as *N*-acetylcysteine or L-cysteine, can augment endogenous H_2_S production and afford protection against hypertension [22,28]. As a sulfur-containing amino acid, taurine can be used for H_2_S synthesis. A previous study reported that its vasodilatory effect might be due to taurine being a substrate for the synthesis of H_2_S to increase CBS and CSE expression [29]. In line with these findings, our data revealed that perinatal taurine treatment enhanced the expression of CBS, CSE, and DAO, as well as H_2_S synthesis, in offspring kidneys. Prior research supported the ability of taurine to enhance the expression of H_2_S-producing enzymes CBS and CSE [30], while little is known about DAO and 3MST. Our results showed that only 3MST expression was not boosted by taurine supplementation. Unlike other H_2_S-producing enzymes, 3MST is located in the mitochondria and catalyzes the production of H_2_S with 3-mercaptopyruvate as the donor of sulfur [31]. Further studies are required to investigate whether taurine differentially regulates H_2_S-producing enzyme expression for such reasons.

Although the H_2_S signaling pathway was enhanced in the CKD + T group, the expressions of H_2_S-generating enzymes and H_2_S production were not obviously altered in control offspring with taurine exposure. Exogenous H_2_S treatment was shown to reduce BP in spontaneously hypertensive rats (SHRs) but had no effect on normotensive Wistar–Kyoto rats [32]. Thus, H_2_S might counterbalance the vasoconstriction observed during hypertension only in diseased subjects (i.e., SHRs) but not in normotensive controls. As BPs are normal in the T group, this requires no compensatory augmentation of the H_2_S signaling pathway in response to perinatal taurine treatment.

Another beneficial action of taurine against maternal CKD-induced hypertension could be attributed to a rebalanced RAAS. We observed that maternal CKD-primed offspring hypertension coincided with aberrant activation of the RAAS. This was in line with previous studies showing that renal programming-induced hypertension and kidney disease are related to aberrant RAAS activation [20]. Taurine has shown benefits to not only established but also developed hypertension via inhibition of the RAAS [33,34]. Our study went one step further to show that perinatal use of taurine can protect offspring from hypertension along with restoring the expression of renin, AGT, ACE, and AT1R induced by maternal CKD.

Notably, perinatal taurine exposure likely programs the RAAS in control offspring, too. Prior research reported that perinatal taurine supplementation did not affect most cardiovascular and metabolic parameters in control offspring [35], while taurine exposure was shown to increase BP in adult male rat offspring; this effect is gender specific [36]. Nevertheless, our data revealed that BP did not differ between the control and taurine-exposed offspring. Such a discrepancy may be due to sex, age, strain, and method of measuring BP. Nevertheless, perinatal taurine supplementation activated the RAAS in control offspring before the appearance of hypertension, suggesting that the perinatal taurine status may enhance the pressor effect and predispose control offspring to develop hypertension in later life. As taurine intake during gestation may program hypertension in the offspring of mothers with normal pregnancies, perinatal taurine supplementation should only be used for indicated cases, but not as a usual dietary supplement in healthy pregnancy.

Since oxidative stress is implicated in the pathogenesis of CKD and taurine has antioxidant properties, it is plausible that the antihypertensive effect of taurine is related to blunting oxidative stress, known to directly quench NO [37]. Nevertheless, conflicting with prior work reporting that the beneficial effects of taurine involve regulation of the NO system [11,12], we found that perinatal taurine supplementation had neglectable effects on plasma NO-related parameters in adult offspring.

The protective mechanisms by which perinatal taurine treatment protects offspring hypertension complicated by maternal CKD are also connected with alterations in gut microbiota from the present study. Regarding gut microbiota, taurine has a protective impact on the host, serves as an energy source for microbes, defends against pathogens, and controls bacterial colonization [38].

*Bifidobacterium* spp. have been regarded as probiotic microorganisms because of their beneficial effects on gut health [39]. Based on our data, taurine administration which restores maternal CKD-induced reduction of *Bifidobacterium* levels may be attributed to its probiotic ability to avert hypertension. Probiotics have a beneficial effect on human health and they are attributed to the production of short-chain fatty acids (SCFAs) [40]. Microbial-derived SCFAs are known to play a key role in BP regulation [41]. Propionate is one of the dominant SCFAs. Previously, we reported that perinatal propionate supplementation protected rat offspring against hypertension programmed by maternal CKD [42]. As the dietary addition of taurine was reported to increase the production of SCFAs [43], additional studies are needed to assess whether SCFAs contribute to the protective action of taurine.

Consistent with research in hypertensive people and animals [44,45,46], we observed that BP was negatively correlated with a high abundance of the genera *Romboutsia*, *Ruminococcus*, *Asteroleplasma*, and *Dehalobacterium*, while it was positively associated with the augmented abundance of *Erisipelactoclostridium*. Also, we found that the species *Ligilactobacillus murinus* and the genus and class to which it belongs were depleted in the CKD group. Our finding ties well with prior research showing that a low abundance of the species *Ligilactobacillus murinus* correlates with hypertension [47]. Our results raise the possibility that the beneficial action of taurine on the developmental origins of hypertension may be related to its capability to alter hypertension-related taxa. In addition, perinatally taurine-exposed offspring exhibited higher proportions of the genera *Alistipes*, *Muribaculum*, and *Turicibacter*. These bacteria are known to process the abundant taurine-conjugated bile acids [38,48]. As disrupted bile acid signaling as a putative mechanism underlying the dysbiotic gut microbiota contributes to hypertension [49], how taurine regulates these microbes and microbial-derived bile acids upon its protective effects is worthy of further evaluation.

Limitations of the current study include a lack of analysis of gut microbiota-derived metabolites in dams and offspring. Given the complex crosstalk between taurine and gut microbiota metabolism [14], the protective role of perinatal taurine supplementation comes from which microbial metabolites (e.g., SCFAs) deserve further clarification. Second, in the current study, only male offspring were enrolled. Whether sex differences occur in the reprogramming effects of taurine needs further clarification. Third, we mainly focused on the kidney in the current study. We cannot exclude the possibility that the protective action of taurine may be specific to other organ systems involved in controlling BP. Fourth, all offspring were sacrificed at 12 weeks of age in the current study. Extended follow-up may provide information on long-term effects, such as the development of hypertension in taurine-exposed control offspring or the life prognosis of four groups. Last, prior work indicated that taurine exerts multiple roles to confer protection against oxidant stress [10,11]. Despite perturbations of H_2_S and NO as contributors to oxidative stress, whether the protective actions of taurine are attributed to other components of oxidative stress deserves further clarification.

## 5. Conclusions

In conclusion, perinatal taurine administration has several protective effects on maternal CKD-induced offspring hypertension, covering the augmentation of the H_2_S system, the rebalancing of the RAAS, and alterations in the gut microbiota. Our findings not only deepen our understanding of the mechanisms behind hypertension of developmental origins but also provide potential therapeutic targets for renal programming-related diseases. Hereafter, taurine-based foods or drugs will be expected to be valuable for the purposes of optimizing global kidney health.

## Figures and Tables

**Table 1:** PCR primers.

Gene	Gene Accession No	Forward	Reverse
Renin	J02941.1	5 aacattaccagggcaactttcact 3	5 acccccttcatggtgatctg 3
PRR	AB188298.1	5 gaggcagtgaccctcaacat 3	5 ccctcctcacacaacaaggt 3
AGT	XM_032887807.1	5 gcccaggtcgcgatgat 3	5 tgtacaagatgctgagtgaggcaa 3
ACE	U03734.1	5 caccggcaaggtctgctt 3	5 cttggcatagtttcgtgaggaa 3
AT1R	NM_030985.4	5 gctgggcaacgagtttgtct 3	5 cagtccttcagctggatcttca 3
CSE	NM_017074.2	5 cgcacaaattgtccacaaac 3	5 gctctgtccttctcaggcac 3
CBS	NM_012522.2	5 atgctgcagaaaggcttcat 3	5 gtggaaaccagtcggtgtct 3
DAO	NM_053626.1	5 ccctttctggaaaagcacag 3	5 ctcctctcaccacctcttcg 3
3MST	NM_138843.2	5 ggctcagtaaacatcccattc 3	5 tgtccttcacagggtcttcc 3
R18S	X01117	5 gccgcggtaattccagctcca 3	5 cccgcccgctcccaagatc 3

**Table 2:** Weight, BP, and kidney function in 12-week-old offspring.

Groups	C	CKD	T	CKDT
Body weight, g	280 ± 5	281 ± 9	308 ± 9	269 ± 9 *^,#^
Left kidney weight, g	1.27 ± 0.029	1.49 ± 0.057 *	1.30 ± 0.032 ^#^	1.19 ± 0.034 *^,#^
Left kidney weight/body weight	0.046 ± 0.001	0.053 ± 0.001 *	0.042 ± 0.001 ^#^	0.044 ± 0.002 ^#^
Creatinine, μM	1.38 ± 0.51	1.29 ± 0.29	1.45 ± 0.52	1.37 ± 0.61
Systolic blood pressure, mmHg	131 ± 1	148 ± 2 *	131 ± 1 ^#^	132 ± 1 ^#^
Diastolic blood pressure, mmHg	88 ± 1	101 ± 2 *	86 ± 2 ^#^	91 ± 2 ^#^
Mean arterial pressure, mmHg	102 ± 1	117 ± 2 *	101 ± 2 ^#^	104 ± 2 ^#^

*n* = 8/group; * *p* < 0.05 vs. C; # *p* < 0.05 vs. CKD. C = control offspring rats; CKD = adenine-exposed offspring rats; T = offspring rats born to dams which received taurine; CKDT = offspring rats born to adenine-treated dams which received taurine.

**Table 3:** Plasma concentrations of NO-related parameters in offspring at 12 weeks of age.

Groups	C	CKD	T	CKDT
L-citrulline, μM	56.6 ± 4.1	50.9 ± 1.3	59.8 ± 2.4	58.5 ± 2.6
L-arginine, μM	179.0 ± 6.3	161.6 ± 3.4	164.5 ± 9.1	166.3 ± 3.4
ADMA, μM	1.71 ± 0.07	2.02 ± 0.09	1.85 ± 0.08	2.18 ± 0.06
SDMA, μM	1.77 ± 0.07	1.62 ± 0.10	1.87 ± 0.04	2.03 ± 0.1
L-arginine to ADMA ratio, μM/μM	113.7 ± 6.0	75.2 ± 4.1	95.6 ± 7.6	72.4 ± 1.8

*n* = 8/group. C = control offspring rats; CKD = adenine-exposed offspring rats; T = offspring rats born to dams which received taurine; CKDT = offspring rats born to adenine-treated dams which received taurine.

## Data Availability

Data are contained within the article.
